# Data-driven analysis of photocatalytic degradation of pararosaniline using green synthesized SrO nanoparticles

**DOI:** 10.1016/j.dib.2025.112096

**Published:** 2025-09-20

**Authors:** Neelam Patil Radhika, Malini S, Shobha G, Kalyan Raj, Shylaja K R, Abhishek Appaji

**Affiliations:** a Department of Chemistry, K S Institute of Technology, Bengaluru, India; b Department of Chemistry, B.M.S. College of Engineering, Bengaluru, India; c Department of Applied science and Humanities, K S Institute of technology, Bengaluru, India; d Department of Medical Electronics Engineering, B.M.S. College of Engineering, Bengaluru, India; e University Eye Clinic Maastricht, Maastricht University, Maastricht, the Netherlands

**Keywords:** Green synthesis, Strontium oxide nanoparticles, Leucas aspera, Pararosaniline, Photocatalytic degradation

## Abstract

Pararosaniline is an extensively used carcinogenic dye whose presence in waterbodies poses serious health risk and hence its photocatalytic degradation is an environmentally relevant dataset. The data reported here highlights the synthesis, characterization and kinetics involved in photocatalytic behaviour of SrO during the degradation of cationic triarylmethane dye pararosaniline. SrO nanoparticles were synthesized by using Plant extract as green fuel from Leucas Aspera and characterized by X-ray diffractometers (XRD), Field emission scanning electron microscopes (FESEM), Fourier transform infrared (FTIR), and UV/vis spectrometer. XRD confirmed the crystalline character of SrO matching with JCPDS database card no. 06–520, FESEM shows the presence of nano-rod like structure with rounded edges having average particle size between 23.20nm–95 nm and FTIR confirming the stretching and bending vibrations of Sr-O bond. The Degradation of pararosaniline was spectrophotometrically examined in the presence of sun light by varying catalyst concentration, dye dosage and pH within 60 min. The highest degradation efficiency 89 % of Pararosaniline was obtained at pH=12 with concentration of catalyst 50 mg and concentration of dye 10 ppm. These findings show the potential application of synthesized SrO nanoparticles for environmental remediation.

Specifications Table

**Table:** 

Subject	Engineering & Materials science
Specific subject area	*Photocatalytic degradation for Environmental remediation*
Type of data	Table, Graph, Figure, Image
How data were acquired	SrO Nanoparticles was prepared using green combustion synthesis at 600 °C, in which *Leucas aspera* leaf extract as green fuel and strontium nitrate are precursors. Physicochemical characterizations were carried out by X-ray diffractometer (XRD), Field emission scanning electron microscope (FESEM), Ultraviolet spectrometer (UV) and Fourier Transform Infrared (FTIR) spectrometer. The optimised photocatalytic behaviour of SrO was explored by varying the concentration of dye and catalyst at various pH which indicated a pseudo first order kinetics.
Data format	Raw and Analyzed
Parameters for data collection	XRD was operated in the range of40-kV voltage with 30-mA current, Diffraction patterns were scanned between 20 and 90° at a scan rate of 0.05°/min. FTIR spectra were collected in wavenumber range 4000–450 cm ^−1^ at a resolution of 4 cm −1 using KBr pellets. FESEM images were collected at accelerating voltage of 15 kV with a magnification of 500 × magnification. The kinetics of UV degradation of Pararosaniline dye under sunlight for a duration of 60 min was analysed for three cycles which indicates a good photostability.
Description of data collection	The XRD Devise (X'Pert Pro Materials Research diffractometer system) was used to gather XRD patterns, and the Carl Zeiss Field Emission Scanning Electron Microscope (FESEM) was used to evaluate the morphology of SrO nanoparticles. UV spectrum (Shimadzu UV- 1700 PharmaSpec double beam UV–Vis Spectrophotometer) and FTIR spectra (Shimadzu FTIR-8400S) were generated with the aid of KBr pellets in the wavenumber range of 4000–450 cm−1 at a resolution of 4 cm−1. Under direct sunlight, the photocatalytic degradation of Pararosaniline, a polyacrylonitrile fibre dye was examined using a UV/vis spectrophotometer.
Data source location	Department of Chemistry, K.S. Institute of Technology, Bangalore Department of Chemistry, BMS College of Engineering, Bangalore.
Data accessibility	Data are available with in this article.
Related research article	None

## 1 Value of the Data

• Nano SrO is previously reported to be nontoxic, possess excellent photocatalytic activity towards toxic dyes and hence is analyzed for its catalytic degradation potential towards Pararosaniline.
• Given the need for sustainable photocatalysts towards toxic dyes that are widely used, the current data provides useful insight for degradation of Pararosaniline a highly toxic Triaryl methane dye.
• The utilization of low-cost plant extract as biofuel not only provides a green route but also excludes sophisticated equipment for preparing nano SrO.
• The data furnished here may help to degrade many other common dyes acting as pollutants in water bodies not necessarily limited to usage of alkaline earth metal oxide based nano photocatalysts.
• The current analysis can be extended to more efficient composites of SrO along with optimization of concentration of catalyst, dye, and pH to achieve 100 % degradation will be the focal point of further research.
• Although considerable reports on photocatalytic activity of nano oxides are evident, this is the first report on nano SrO aided degradation of Pararosaniline dye.
• This data is highly beneficial in providing insights into an eco-friendly dye remediation to academicians, industrialists and regulatory authorities.

## 2 Background

Pararosaniline is known to pose high health risks due to its severe carcinogenicity [1] and hence its degradation is an environmentally relevant process. Pioneering results shown by nano ZnO [2], TANI/Cu_2_O/Ag nanocomposite [3] has established photocatalytic route as an economic and effective route to degrade Pararosaniline. In this regard, Nano SrO is an emerging photocatalyst investigated for its activity on Methylene blue along with carmine [4], Malachite green along with benzene [5] and common organic pollutants [6]. Except for the leaf extract driven synthesis of SrO [7], no prior reports on photodegradation of Pararosaniline using green nano SrO is reported to the best of our knowledge and hence this study qualifies to be an environmentally significant data. *Leucas Aspera* was chosen to be plant source considering the huge success of it as a fuel while preparing Fe_2_O_3_ [8] and Ag [9] nano catalysts towards photocatalytic dye degradation. In the present study a measured quantity of nano metal oxide and the dye are made to react in a dispersed medium under sunlight and monitored at regular intervals using UV spectrophotometer to note the decrease in absorbance at characteristic wavelength. The dataset obtained is utilized to study the kinetics and optimize the conditions which adds value to the existing knowledge on dye degradation.

## 3 Data Description

**Fig. 1:**
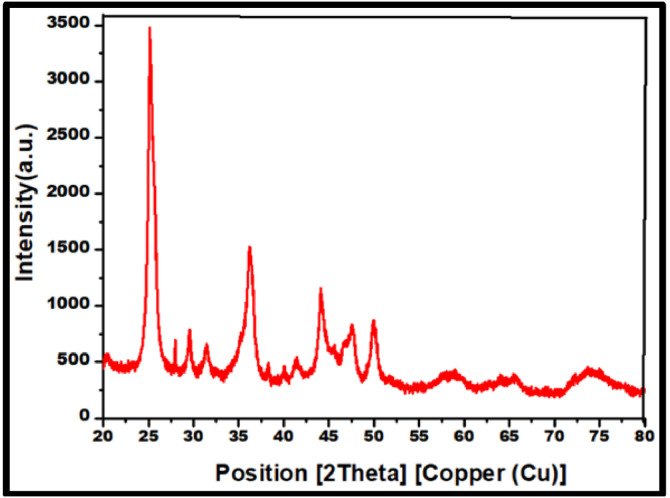
X-ray diffraction pattern of SrO nano particles.

X-ray diffraction analysis used to analyze the crystalline character of the prepared SrO nanoparticles in Fig. 1 shows 2̟Ɵ diffraction peaks at 25.11°, 29.6°,36.16°, 49.86°, 58.56, and 74.16 which correspond to the crystal planes (202), (111), (200), (220), (311) and (440) respectively. The crystallographic phase of the sample is confirmed by comparing the 2θ value of each peak and its related Miller indices with standard references from the JCPDS database (card no. 06–520) which is similar to previously reported data [[10], [11], [12]]. The development of strong, sharp peaks and the absence of additional peaks confirm the phase purity and crystalline character. The average crystallite size of the sample was approximately 23 nm calculated using Debye Sherer equation. However, crystallite size distribution studies which will be undertaken in the future, will provide clarity on crystallite size distribution.

**Fig. 2:**
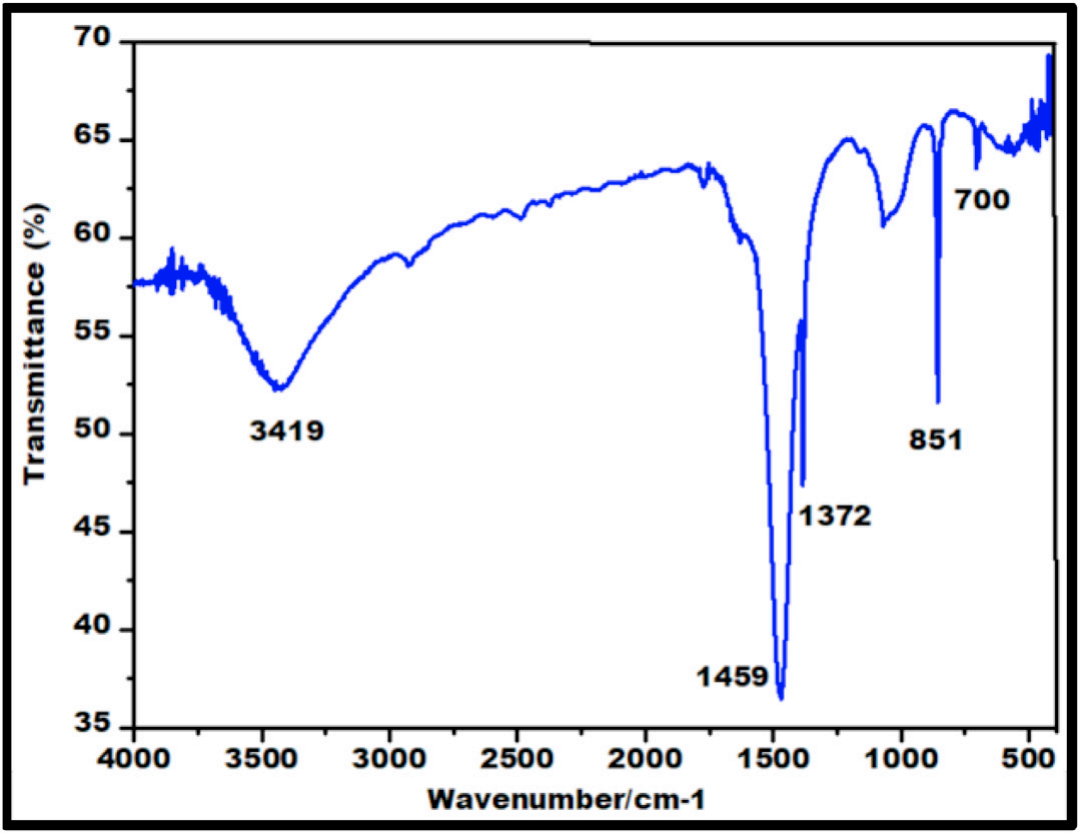
FT-IR spectra of SrO nanoparticles.

**Fig. 3:**
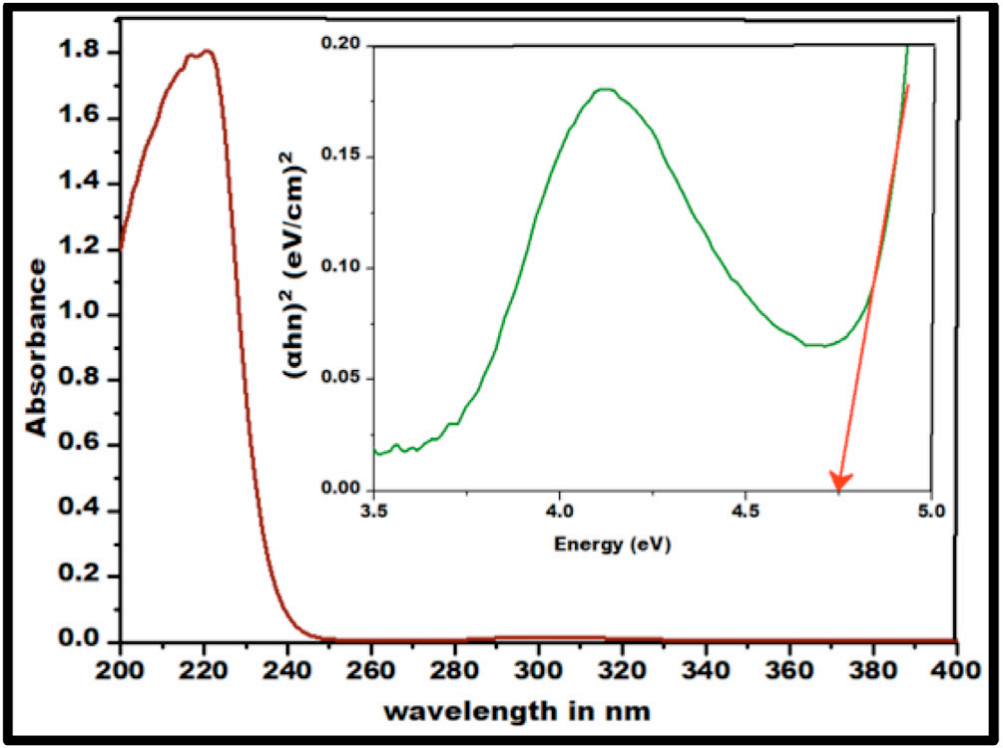
UV–visible absorption spectra (Inset Tauc’s plot) for SrO nanoparticles.

**Fig. 4:**
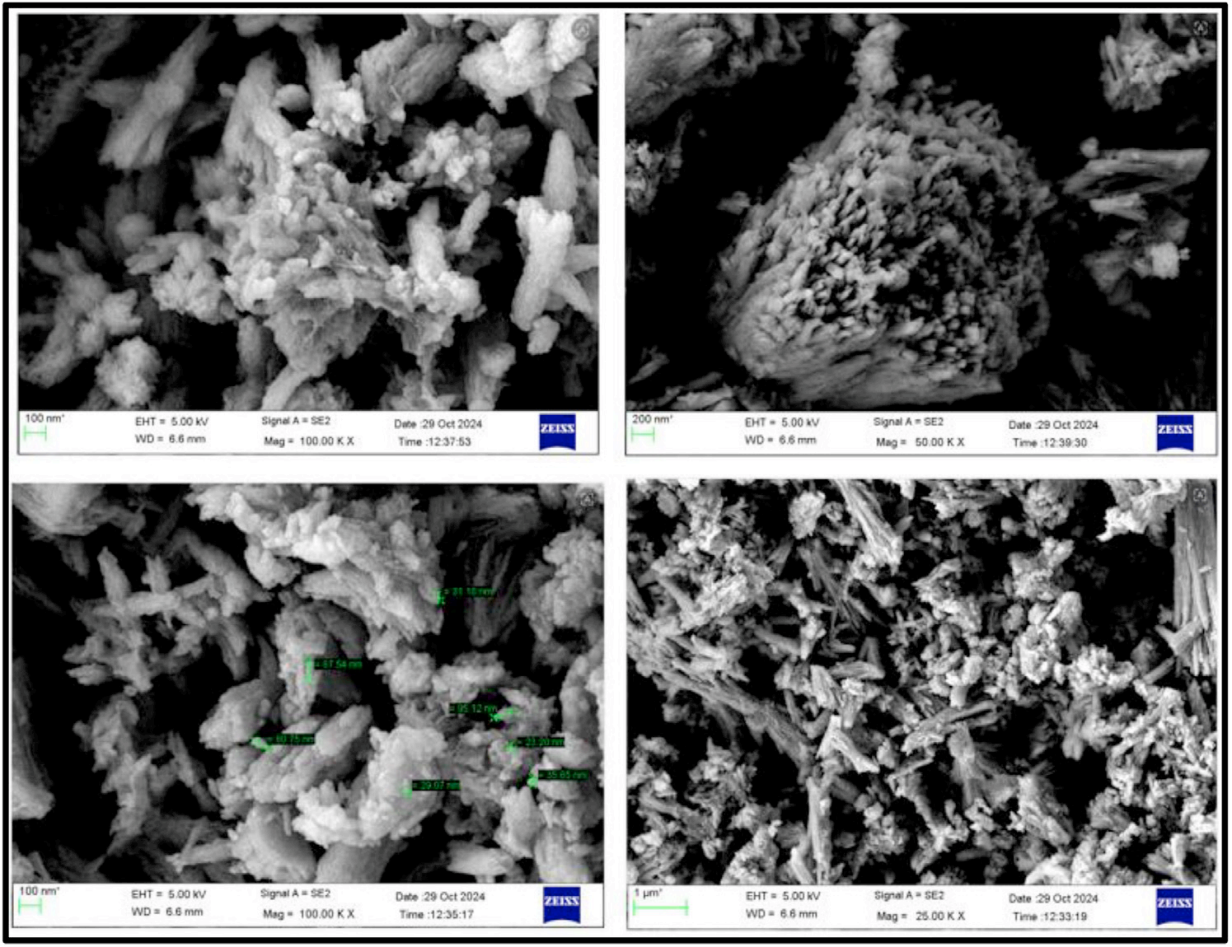
FESEM images of nano SrO.

**Fig. 5:**
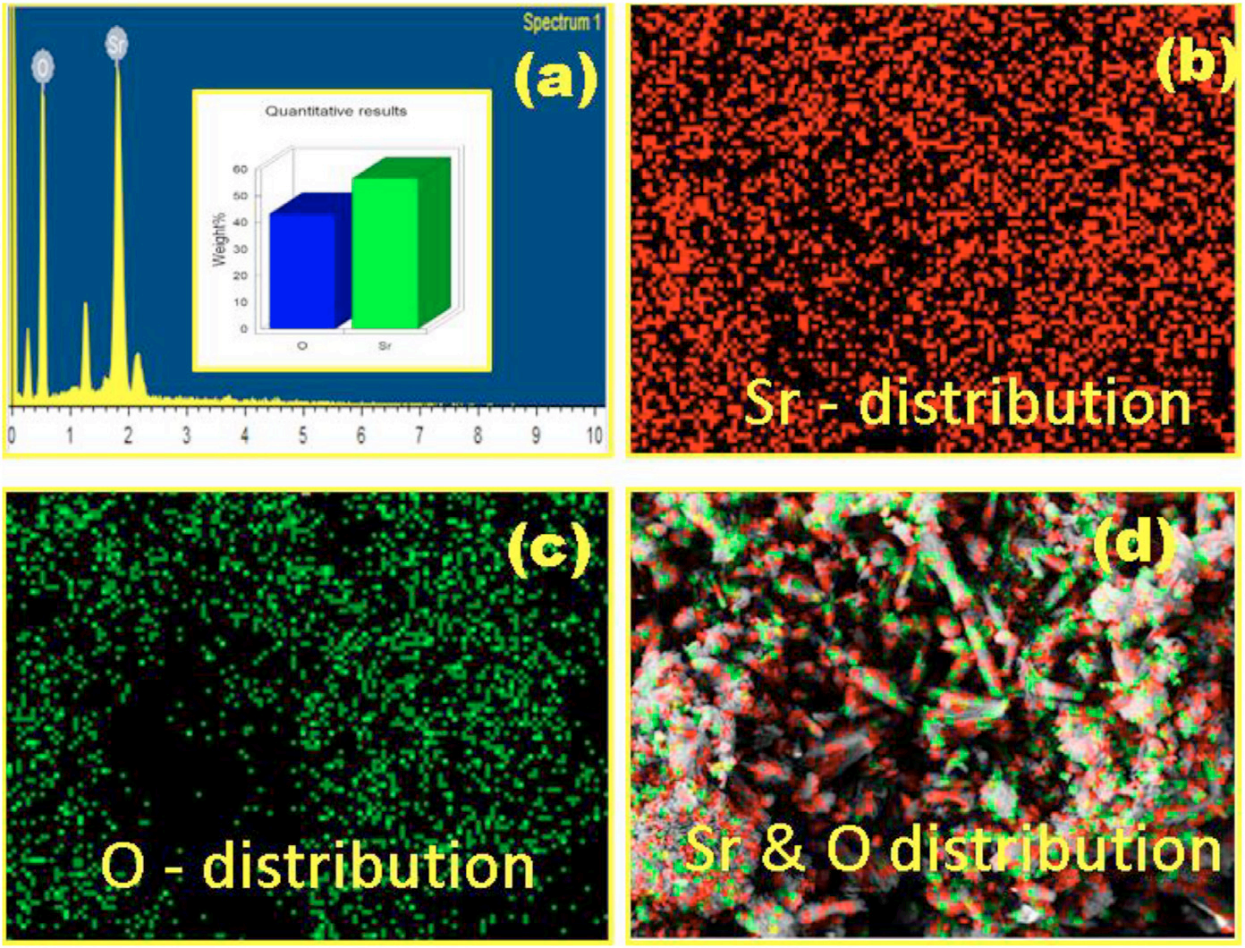
Elemental analysis of synthesized SrO nanoparticles (a)EDAX spectrum (b-d) mapping images.

FTIR peaks obtained in the range 4000–400 cm^−1^ in Fig. 2 displays the characteristic peaks of SrO nanoparticles. The presence of Sr–O is confirmed by the peaks at 856.42 and 700.18 cm⁻¹, which correlates with the bending vibrations and a peak at 1372cm^−1^ is due to assymetric stretching vibration of the metal–oxygen bond which matches with the early research [13]. The green synthesized SrO nanoparticle were confirmed by UV visible Spectrum shown in between 200 nm to 400 nm using a spectrophotometer in which maximum peak was observed at 220 nm shown in Fig. 3 indicating the successful synthesis of SrO nanoparticles as reported previously [14]. Also, a linear curve provided by the plot of (αhν)^2^ against hν on extrapolation to zero indicated a bandgap of 4.7 eV correlating to that formerly reported [15]. The FESEM presented in Fig. 4 was used to assess Nano rod-like structures in different sizes having a rough surface [16,17] were evenly distributed with noticeable aggregation [11,18]. However, specific surface area analysis can be obtained through BET data which will be undertaken in the future. Energy X-ray dispersive analysis (EDAX) displayed in Fig. 5 is utilized to evaluate the elements in the SrO nanoparticles both quantitatively and qualitatively which confirmed the presence of strontium (Sr) and oxygen (O), with a composition of 56.63 % and 43.37 %, respectively. These findings indicate the successful conversion of strontium ions into elemental strontium and oxygen, validating the formation of SrO nanostructures [11].

## 4 Experimental Design, Materials and Methods

### 4.1 Collection and preparation of Leucas aspera leaf extract

*Leucas aspera* weed are found growing as dense clumps along the roadsides of Bangalore, Karnataka, India. *Leucas aspera* leaves were separated from plants and thoroughly cleaned to remove any dust, plants were thoroughly cleansed with tap water and then they were thoroughly rinsed in deionized water. The washed leaves were shade dried in room temperature before being ground into a fine powder with a mechanical blender. A 1:50 (g/v) ratio of plant powder to deionized water was used to create the aqueous solution. The obtained mixture was filtered through Whatman filter paper and kept at 4 °C to utilize for further use.

### 4.2 Synthesis of strontium nanoparticles (SrO NPs)

**Fig. 6:**
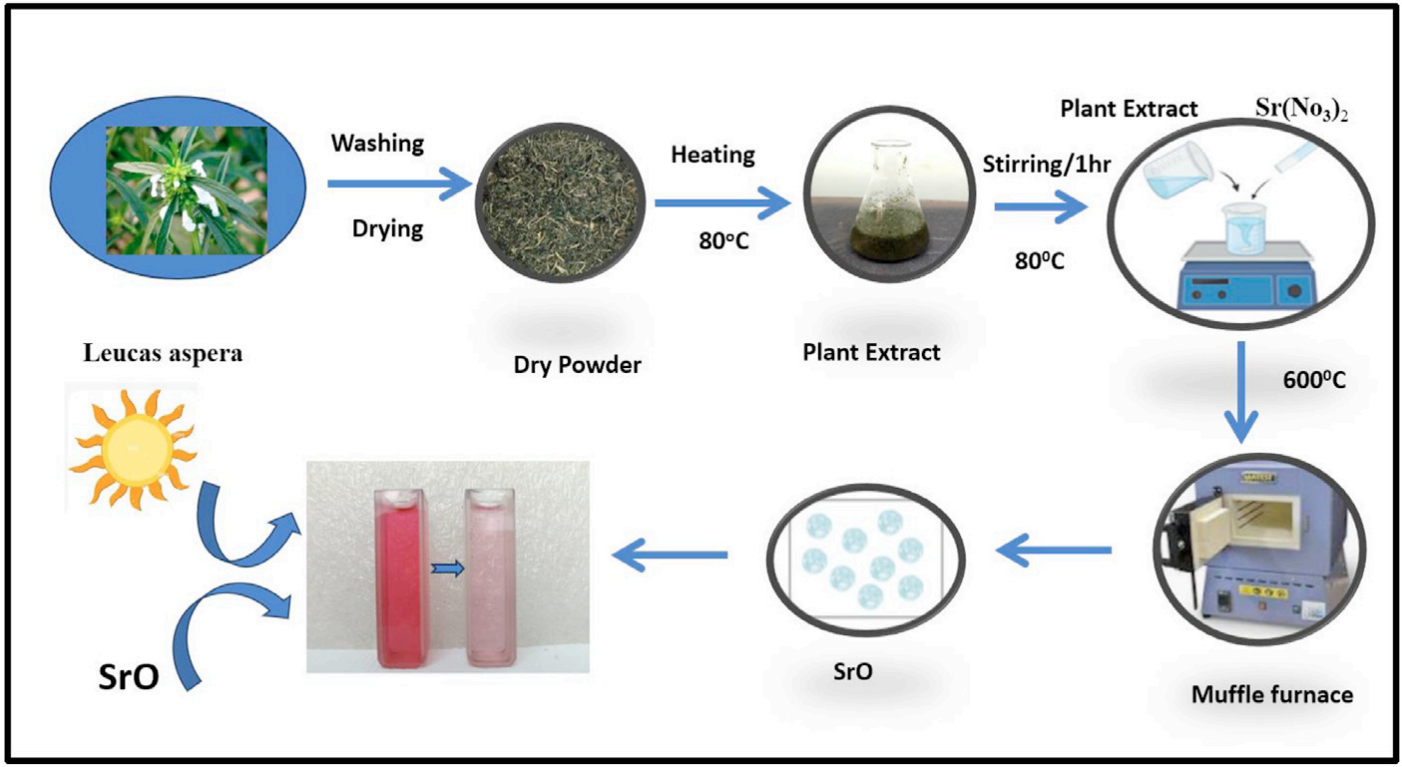
Schematic representation of synthesis of SrO Nanoparticles followed by degradation of Pararosaniline.

To synthesize SrO nanoparticles, 90 mL of strontium nitrate (0.01 M) was mixed with 20 mL of aqueous leaf extract. The mixture was maintained on magnetic stirrer at 80 °C for 30 min. The development of nanoparticles was indicated by the solution color change. To remove any remaining biological molecules, the final Nano-colloidal solution was subjected to centrifugal process at 8000 rpm for 25 min in a Remi Research Centrifuge. The last pellet was gathered, rinsed with ethanol to get rid of ionic contaminants after being washed with deionized water till the filtrate became clear [14]. The resulting white precipitate was calcinated at 600 °C preserved for later use. The synthesis of NPs is displayed in the Fig. 6.

The evaluated rate constant during photocatalytic degradation with variance of concentration of catalyst, dye and pH is summarized in Table 1, Table 2, Table 3.

**Table 1:** Reaction rate constants (K) for pseudo first order reaction and coefficient of determination (R^2^) obtained for photodegradation of Pararosaniline using different concentrations of SrO Nanoparticles.

SrO concentration	R^2^ value	K (min ^−1^)
50	0.974	0.0254
100	0.9116	0.018
150	0.9277	0.0093

**Table 2:** Reaction rate constants (K) for pseudo first order reaction and coefficient of determination (R^2^) obtained for photodegradation of Pararosaniline using different concentrations Pararosaniline.

Pararosaniline	R^2^ value	K (min ^−1^)
10	0.9545	0.028
20	0.9416	0.011
30	0.9388	0.009

**Table 3:** Reaction rate constants (K) for pseudo first order reaction and coefficient of determination (R^2^) obtained for photodegradation of Pararosaniline using different pH.

pH	R^2^ value	K (min ^−1^)
12	0.9057	0.0364
7	0.8352	0.0147
4	0.7669	0.017

### 4.3 **Photocatalytic degradation of pararosaniline using** SrO

*Leucas aspera* facilitated SrO nanoparticles photocatalytic activity was evaluated against pararosaniline dye while exposed to sun light. To achieve this, a polluted water sample with 10 mg/L pararosaniline molecules was mixed with the SrO catalyst and left in the dark for 20 min to approach the adsorption–desorption equilibrium. Next, the dark-conditioned samples were removed and placed directly under sunlight focused by a convex lens during 12.30 noon to 1.30 pm in the month of April with solar radiometer displaying 0.750 kW *m*^−2^ of radiation intensity.

**Fig. 7:**
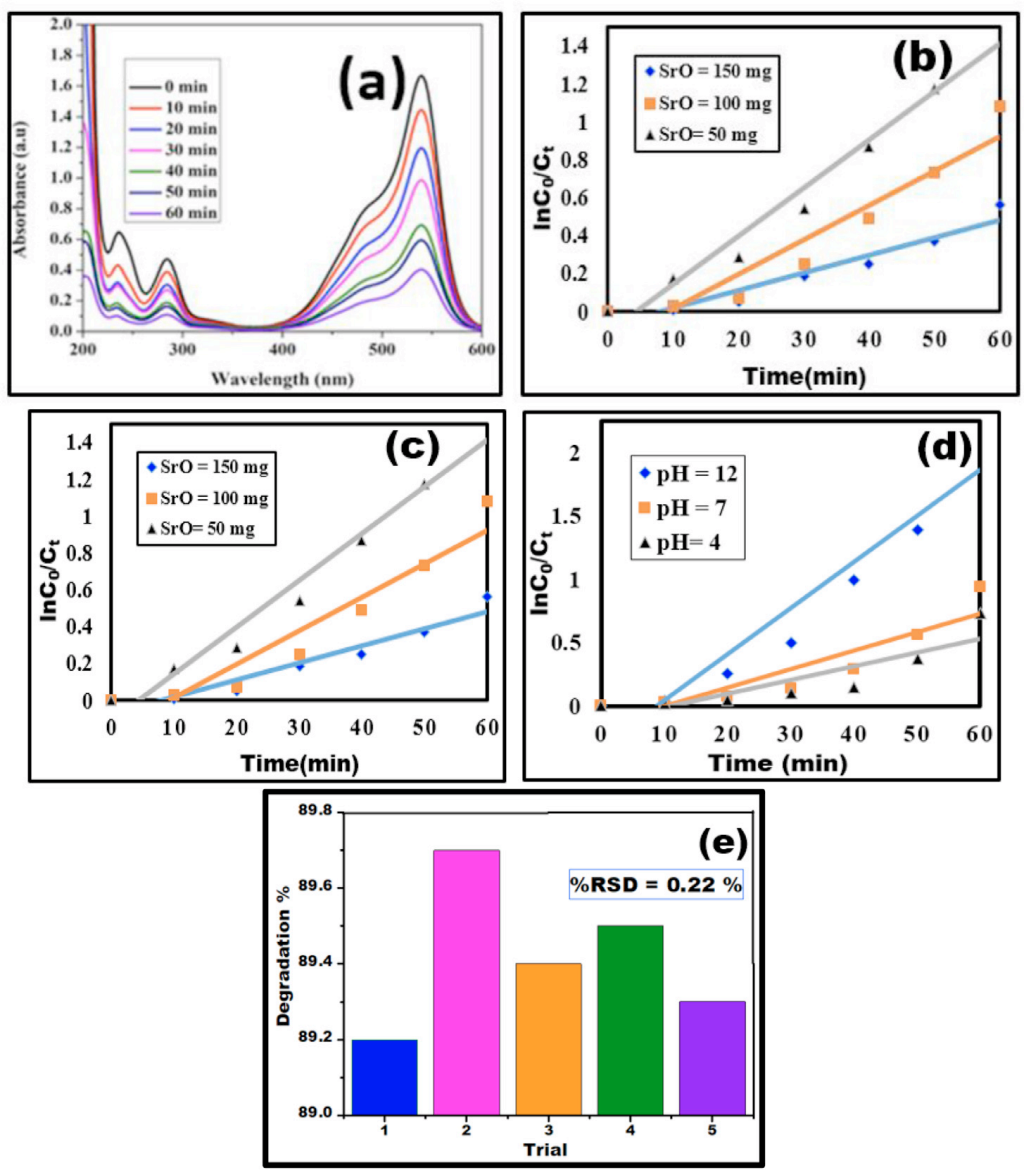
(a) UV spectrum showing photocatalytic degradation activity of SrO nanoparticles at different time intervals of sunlight irradiation (b) Pseudo first order kinetic fit by varying concentration of SrO (c) Pseudo first order kinetic fit by varying concentration of Pararosaniline (d) variation of pH with SrO (50 mg/L) & PA (10 mg/L) (e) Error bars for degradation efficiency.

A test sample was taken from the solution at regular intervals of 10 min while the solution was exposed to sunlight for 60 min as reported previously [19]. The SrO catalyst was extracted from the solution by centrifuging the obtained samples. Using equation (1), the degradation efficiency was determined from the UV–Vis spectra:

(1) $$Dye\;degradation\;\left(\%\right)=1-C_{t}/C_{0}*100$$

where C_0_ is the initial concentration of the pararosaniline dye solution before it was exposed to light, and C_t_ represents the concentration of the dye solution at various time intervals after being exposed to sunlight. The experiment was conducted for five cycles under identical conditions to ensure the accuracy of the degrading efficiency. An error bar in Fig. 7(e) represents the efficiency of 89.2, 89.7, 89.4, 89.5, 89.3 across five cycles resulting in a mean of 89.42 % and a low value RSD of 0.22 % indicating a highly consistent quantification.

### 4.4 Reaction scheme

The likely reaction scheme for the photocatalytic degradation of Pararosaniline dye can be depicted as follows where SrO in response to light reaches an excited state which on relaxing releases an electron and a hole. The hole oxidizes hydroxide ions to hydroxyl radicals while the electron in the conduction band reduces molecular oxygen to superoxide anion radicals that cleaves water to generate hydroxide ion and thereby facilitate the base catalyzed degradation of Pararosaniline as inferred from Fig. 7(d).

The base catalyzed degradation is predicted to be initiated by loss of protons in amino groups that destabilizes the resonance in the chromophore and trigger the nucleophilic attack of hydroxide ion on the central carbon of the triphenylmethane core. The resonance system is disrupted with subsequent cleavage of the aryl–central carbon bonds leading to the formation of smaller substituted benzene derivatives that are less toxic than Pararosaniline.

However, isolation followed by spectroscopic and chromatographic studies would elucidate the exact nature, structure and confirm the formation mechanism of the degradation products which will be undertaken in the future by the authors.

Photocatalytic performance is influenced by several variables, including pH, catalyst dosage, dye concentration, and degradation time. To optimize these parameters, one variable was kept constant in this study while the others were changed. Decolorization was evaluated with catalyst doses ranging from 50 mg/L to 150 mg/L at intervals of 0–60 min in the presence of sunlight, while maintaining a fixed PA dye concentration of 10 mg/L. Fig. 7**(a)** displays the pararosaniline catalyst degradation efficiency. At 60 min, the highest percentage of decolorization observed when the catalyst concentration was 50.0 mg/L. The rate constant values and R^2^ values for degradation of pararosaniline by varying SrO concentration were displayed in Table 1. Similarly rate constant values and R^2^ values by varying pararosaniline concentrations and at different pH levels were displayed in Table 2, Table 3. From this data it indicates that highest degradation of pararosaniline by using SrO nanocatalyst at pH 12 is 89 %.

### 4.5 Effect of catalyst dosage

The influence of catalyst dosage on dye degradation was examined by varying the catalyst SrO (50 mg/L, 100 mg/L and 100 mg/L), while keeping other parameters such as dye concentration constant Fig. 7(b) and results showed that dye degradation efficiency was highest at 50 mg/L and hence was fixed as an optimal dose. This trend is typical in heterogeneous photocatalysis, where an increase in catalyst dosage leads to a greater number of active sites [20] on the catalyst surface, enhancing the formation of hydroxyl radicals (●OH) and ions that are responsible for dye degradation [21]. However, when the catalyst dose exceeded 50 mg/L a decline in degradation efficiency was noted. This decrease is attributed to increased turbidity in the solution, which hampers the penetration of UV light, thereby limiting the photocatalytic reaction.

### 4.6 Effect of dye dosage

The degradation of Pararosaniline dye was investigated at varying initial concentrations of 10 mg/L, 20 mg/L and 30 mg/L of dye, catalyst dosage was kept constant as in Fig. 7(c). The highest degradation efficiencies observed for 10 mg/L Pararosaniline dye concentration after 60 min. However, as the initial dye concentration increased, the degradation efficiency declined. This reduction is attributed to the increased number of dye molecules, which limits light penetration into the solution and reduces the photon flux reaching the catalyst surface [22]. As a consequence, the generation of reactive species like hydroxyl and superoxide radicals diminishes.Furthermore, at higher concentrations, active adsorption sites on the catalyst surface become saturated, further decreasing degradation efficiency.

### 4.7 Effect of pH

**Fig. 8:**
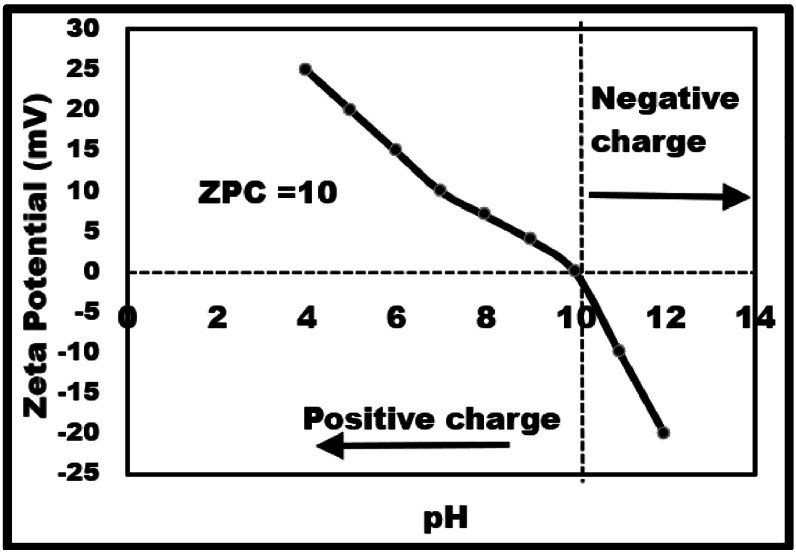
Zeta potential analysis of SrO at different pH.

The influence of pH on pararosaniline dye degradation was studied by adjusting the pH values from 4 to 12, while keeping all other experimental conditions constant as in Fig. 7(d). The results revealed that dye degradation efficiency raised with increasing pH and highest degradation was observed at an elevated pH of 12. Further, from Zeta potential measurements represented in Fig. 8 relates pH and surface charge. It is evident that Zeta potential progressively decreases with increasing pH, reaches zero or “zero point of charge” at pH 10 and thereon becomes negative reaching a minimum at pH 12 suggesting an induced surface negativity over the Nano catalyst. Thus, positively charged Pararosaniline dye molecules are electrostatically attracted resulting in an enhanced Pararosaniline pollutant interaction with the catalyst surface [23]. Numerous researchers have highlighted that metal oxide nanoparticles achieve higher degradation efficiency in alkaline environments. [24]

## Ethics Statement

Authors have read and follow the ethical requirements for publication in Data in Brief and confirm that the current work does not involve human subjects, animal experiments, or any data collected from social media platforms

## Credit Author Statement

**Neelam Patil Radhika:** Performed the experiments, Wrote the paper. **Malini S:** Conceived and designed the experiments, Wrote the paper. **Kalyan Raj:** Contributed reagents and materials. **Shobha G:** Interpretation and Data analysis. **Shylaja K. R:** Contributed Analysis tools. **Abhishek Appaji:** Analyzed and interpreted the data.

## Declaration of Competing Interest

The authors declare that they have no known competing financial interests or personal relationships that could have appeared to influence the work reported in this paper.

## Data Availability

IEEE DataPortPhotocatalytic Degradation of Pararosaniline by nano SrO – Experimental Data (Original data)
